# Comparison of the Latarjet Procedure With the Modified Putti-Platt Surgery for Recurrent Anterior Shoulder Dislocation With Respect to Functional Outcome

**DOI:** 10.7759/cureus.57270

**Published:** 2024-03-30

**Authors:** Ihtisham Anjum, Ubaid Ullah, Aimen Fazli Maula, Hamza Haroon, Yaseen Ahmad, Rao E Hassan, Kashan Shahid, Saddam Hussain, Waseem Haider, Rizwan Ullah

**Affiliations:** 1 Orthopaedics and Trauma, Khyber Teaching Hospital Medical Teaching Institution (MTI), Peshawar, PAK; 2 Emergency Medicine, Shrewsbury and Telford Hospital NHS Trust, Shrewsbury, GBR; 3 Surgery, Ayub Teaching Hospital, Abbottabad, PAK

**Keywords:** hill-sachs lesion, shoulder anterior dislocation, recurrent shoulder dislocation, putti-platt, latarjet procedure, bankart’s lesion, shoulder instability

## Abstract

Introduction

Shoulder dislocation is the most common injury encountered in orthopedic outpatient department. The choice of procedure depends on the expertise of surgeons. The objective of this study was to compare the Latarjet procedure with the modified Putti-Platt surgery for recurrent anterior shoulder dislocation in terms of functional outcomes.

Materials and methods

A quasi-experimental study evaluated 60 patients with recurrent anterior shoulder instability. Patients were assigned to either Latarjet or modified Putti-Platt surgery. Functional outcomes were assessed at six months using the Constant-Murley shoulder score.

Results

This study encompassed 60 patients (mean age: 23.93±5.88 years) undergoing shoulder procedures. Functional outcomes exhibited a majority of 55% excellent, 35% good, 6.7% fair, and 3.3% poor outcomes. No significant differences in functional outcomes were found between the procedures.

Conclusion

Both procedures may be viable options for recurrent shoulder instability. The choice may depend on patient factors and surgeon preference. Further research is needed to refine techniques and identify ideal candidates.

## Introduction

The shoulder joint, inherently unstable due to its shallow socket, is the most commonly dislocated major joint (50% of cases). Anterior dislocations are the most frequent type. Ligaments, particularly the inferior glenohumeral ligament, and the capsule complex primarily stabilize the shoulder [1,2]. Rotator cuff tears, especially in the four muscles surrounding the joint (supraspinatus, infraspinatus, subscapularis, teres minor), can also contribute to instability [3].

Shoulder dislocations are overwhelmingly caused by trauma (95% of cases), with the remaining 5% due to atraumatic factors. Identifying the cause (traumatic vs. atraumatic) is crucial for understanding the underlying reason for the dislocation and guiding treatment decisions. Age at first dislocation is a key predictor of future occurrences. Younger patients (teens) have a significantly higher recurrence rate (around 90%) compared to those over 40 (10-15%) [4].

For the definitive treatment of an unstable shoulder, surgery may be necessary. While arthroscopic techniques are increasingly used, open procedures can achieve similar results. The Putti-Platt procedure, a long-standing open technique, aims to stabilize the joint by tightening the subscapularis muscle and anterior capsule. However, it doesn't address underlying issues like labral tears and can lead to limited external rotation and potential osteoarthritis. Over the years, several modifications have been developed to address these limitations [5].

The Latarjet procedure is a relatively new surgical technique for shoulder instability. A study examined 35 patients who underwent the Latarjet procedure, with 74% (26 patients) having the surgery on their right shoulder. At six months post-surgery, outcomes were rated as excellent in 66% (23 patients), good in 23% (eight patients), fair in 6% (two patients), and poor in 6% (two patients). The study documented significant pain reduction and improved shoulder motion at six months (p-value <0.05) [6].

Another study using the Constant-Murley shoulder score found that 96% (40 patients) of those who underwent the modified Putti-Platt procedure achieved a score exceeding 86 points, indicating an excellent outcome [7].

This study aims to compare the functional outcomes of Latarjet surgery and modified Putti-Platt surgery for recurrent shoulder dislocation. A prior literature review identified no existing studies directly comparing the functional outcomes of these two procedures.

## Materials and methods

The study, conducted at the Department of Orthopedic Surgery, Khyber Teaching Hospital, Peshawar (November 1, 2022-October 31, 2023), employed a quasi-experimental design. Sixty participants were divided into two groups of 30 each. This sample size was calculated using the WHO Sample Size Calculator, considering previous studies reporting a 65.31% excellent outcome rate for Latarjet surgery [6] and 96% for modified Putti-Platt surgery [7], with a 95% confidence interval, 80% power, and 5% margin of error. Non-probability consecutive sampling was used. Patients included had an anterior shoulder dislocation, were 15-40 years old, were of any gender, and were classified as American Society of Anesthesiologists (ASA) Scale I. We excluded patients unwilling to participate, athletes, those with prior shoulder surgery, and individuals with posterior dislocation, atraumatic instability, or general joint laxity (confirmed by clinical history and examination).

Following ethical approval from the Khyber Medical College Institutional Research and Ethical Review Board (IREB) (approval number: 785/DME/KMC), patients with recurrent anterior shoulder dislocation were enrolled according to the inclusion criteria. Written informed consent was obtained, and all participants were thoroughly briefed about the study's purpose. The selected patients were divided into two groups. Group A underwent Latarjet surgery, while Group B received modified Putti-Platt surgery.

A physical examination assessed shoulder stability using sulcus and anterior drawer tests. The examination included the other shoulder and joints to exclude generalized ligament laxity. X-rays (anterior-posterior (AP), axillary, and apical oblique views) were used to visualize bone structures, while MRI scans evaluated for Bankart lesions (labral tears), Hill-Sachs lesions (humeral head defects), and capsule tears.

Both procedures used general anesthesia administered through tracheal intubation and intravenous medications. Patients were positioned supine with a scapular region cushion on the surgical side.

The Latarjet procedure involved a 5-7-cm incision along the deltopectoral interval, extending to the axillary fold. The coracoid process was exposed and the acromioclavicular ligament was identified and cut, and a cuff was left on the coracoid for later repair. The pectoralis minor tendon and medial coracoid tissue were released to facilitate positioning against the glenoid. The axillary and musculocutaneous nerves were protected throughout the procedure. A 90° oscillating saw harvested the coracoid bone graft, preserving nerves and blood supply. The procedure then focused on glenoid exposure: splitting the subscapularis muscle, performing a capsulotomy, and preparing the glenoid bed. The coracoid graft was positioned against the glenoid and secured with a single malleolar screw. The capsular repair was done using the remaining acromioclavicular ligament, followed by repair of the subscapularis muscle.

The modified Putti-Platt procedure involved a 7-cm incision along the deltopectoral groove, starting near the coracoid process. The subscapularis tendon was exposed and then cut 1-2 cm from its attachment point, and the capsule was opened for glenoid visualization using a Fukuda retractor. If a detached labrum was found, it was reattached to the glenoid rim and capsule with suture anchors. Any capsular tears were repaired as well. The subscapularis muscle was tightened by transferring its medial portion under the lateral portion, creating a capsular overlap. To prevent excessive tightness and limited motion, the surgeon assessed the repaired structures with the arm in a specific position (elbow bent at 90°, thumb up) and ensured the shoulder could reach a neutral position (0° external rotation). Finally, the lateral subscapularis tendon was attached to the capsule, avoiding suturing it directly to the labrum.

In both groups, a thoracobrachial bandage was applied for 21 days and changed weekly for wound inspection and hygiene. Stitches were removed on the 10th post-operative day. Pain management consisted solely of analgesics. Rehabilitation began on day 22 with passive pendulum exercises, progressing to isometric and active exercises for muscle strengthening and joint mobility restoration. A polysling was used for support until the sixth week. Full range of motion exercises were started after the end of the fourth month. Both groups underwent post-operative shoulder function evaluation at six months using the Constant-Murley score (excellent: 86-100, good: 71-85, fair: 56-70, poor: <56) [8]. This score assesses pain, function, range of motion, and strength. Patient demographics and functional outcomes were recorded on a separate form.

Data analysis was performed using IBM SPSS Statistics for Windows, V. 24.0 (IBM Corp., Armonk, NY). For numerical data like age, surgery time, and hospital stay, means and standard deviations were calculated. Categorical data, including gender, occupation, education, social class, and functional outcomes in both groups, were analyzed using frequencies and percentages. The chi-squared test compared functional outcomes between the groups. Stratification was then performed to assess the influence of age, gender, occupation, education, and social class on functional outcomes. Furthermore, chi-squared tests were applied within each stratum, with a significance level of p<0.05.

## Results

This study enrolled 60 patients with an average age of 23.93±5.88 years. They were divided into two groups: 30 in the Latarjet group (mean age 24.23±6.26 years) and 30 in the modified Putti-Platt group (mean age 23.44±5.72 years). The Latarjet procedure had a longer operative time (1.25±0.5 hours) and hospital stay (2.25±0.57 days) compared to the modified Putti-Platt procedure (0.73±0.32 hours and 2.16±0.61 days, respectively).

The patient demographics were as follows: 70% (n=42) male and 30% (n=18) female; 70% (n=42) employed; and 33.3% (n=20) residing in rural areas. Education levels were 8.3% (n=5) illiterate, 45% (n=27) primary education, and 46.7% (n=28) secondary education or higher. Socioeconomic status was classified as 21.7% (n=13) poor, 40% (n=24) middle class, and 38.3% (n=23) rich. 

The Constant-Murley score assessed functional outcomes at the end of six months of surgery. Patients achieved excellent results in 55% (n=33) of cases, good results in 35% (n=21), fair results in 6.7% (n=4), and poor results in 3.3% (n=2).

There was no statistically significant difference in functional outcomes between the Latarjet and modified Putti-Platt procedures (p>0.05) despite varying percentages of excellent, good, fair, and poor outcomes (Table 1).

**Table 1:** Functional outcomes in both groups The chi-squared test was used with a p-value ≤0.05 significant

Groups	Functional outcomes categories				Total	P-value
	Excellent	Good	Fair	Poor		
Latarjet procedure	15	12	3	0	30	
Modified Putti-Platt procedure	18	9	1	2	30	
Total	33	21	4	2	60	0.296

Social class (Table 2), education level (Table 3), and residence (Table 4) significantly impacted functional outcomes in both procedures (p<0.05). However, gender (Table 5) and employment status (Table 6) did not show any statistically significant associations.

**Table 2:** Comparison of functional outcomes with social class in both groups The chi-squared test was used with a p-value ≤0.05 significant

Groups			Functional outcomes categories				Total	P-value
			Excellent	Good	Fair	Poor		
Latarjet procedure	Social class of the patient	Poor	0	7	3	0	10	
		Middle class	2	5	0	0	7	
		Rich	13	0	0	0	13	
	Total		15	12	3	0	30	<0.001
Modified Putti-Platt procedure	Social class of the patient	Poor	0	0	1	2	3	
		Middle class	10	7	0	0	17	
		Rich	8	2	0	0	10	
	Total		18	9	1	2	30	<0.001

**Table 3:** Comparison of functional outcomes with education status in both groups The chi-squared test was used with a p-value ≤0.05 significant

Groups			Functional outcomes categories				Total	P-value
			Excellent	Good	Fair	Poor		
Latarjet procedure	Education status of the patient	Illiterate	0	1	0	0	1	
		Primary	2	11	2	0	15	
		Secondary and above	13	0	1	0	14	
	Total		15	12	3	0	30	<0.001
Modified Putti-Platt procedure	Education status of the patient	Illiterate	0	1	1	2	4	
		Primary	7	5	0	0	12	
		Secondary and above	11	3	0	0	14	
	Total		18	9	1	2	30	0.001

**Table 4:** Comparison of functional outcomes with residence status in both groups The chi-squared test was used with a p-value ≤0.05 significant

Groups			Functional outcomes categories				Total	P-value
			Excellent	Good	Fair	Poor		
Latarjet procedure	Residence	Rural	0	8	3	0	11	
		Urban	15	4	0	0	19	
	Total		15	12	3	0	30	<0.001
Modified Putti-Platt procedure	Residence	Rural	0	6	1	2	9	
		Urban	18	3	0	0	21	
	Total		18	9	1	2	30	<0.001

**Table 5:** Comparison of gender and function outcomes in both groups The chi-squared test was used with a p-value ≤0.05 significant

Groups			Functional outcomes categories				Total	P-value
			Excellent	Good	Fair	Poor		
Latarjet procedure	Gender of the patient	Male	12	9	3	0	24	
		Female	3	3	0	0	6	
	Total		15	12	3	0	30	0.626
Modified Putti-Platt procedure	Gender of the patient	Male	11	5	1	1	18	
		Female	7	4	0	1	12	
	Total		18	9	1	2	30	0.841

**Table 6:** Comparison of functional outcomes with education status in both groups The chi-squared test was used with a p-value ≤0.05 significant

Groups			Functional outcomes categories				Total	P-value
			Excellent	Good	Fair	Poor		
Latarjet procedure	Occupation	Employed	10	10	3	0	23	0.616
		Unemployed	5	2	1	0	7	
	Total		15	12	3	0	30	
Modified Putti-Platt procedure	Occupation	Employed	11	6	1	1	19	0.846
		Unemployed	7	3	0	1	11	
	Total		18	9	1	2	30	

## Discussion

This study compared two surgical techniques for treating recurrent anterior shoulder dislocation in 60 patients with an average age of 23.93±5.88 years. Our findings on post-operative stability, mobility, and function align with existing research [9,10]. This research suggests a higher risk of recurrent dislocation in younger patients, with a higher incidence in males. Studies have shown that up to 90% of patients with recurrent dislocation may be younger than 20 years old [11]. Additionally, another study reported that 87.5% of patients with shoulder dislocation were under 20, with the remaining patients falling between 20 and 40 years old [10].

The Latarjet procedure offers excellent functional outcomes and low recurrence rates for recurrent shoulder instability [12]. Success hinges on the accurate placement and secure fixation of the coracoid bone graft [13]. This technique aims to reconstruct the vulnerable anteroinferior glenoid, often eroded or fractured in these patients [14]. While open surgery can limit visualization of the glenoid, it may also simplify certain technical aspects of the procedure [15].

The Putti-Platt procedure is often criticized for potentially reducing the range of motion, making it less suitable for young, active patients [16]. Our modified technique addressed these concerns through two key changes. First, we avoided suturing the lateral subscapularis tendon directly to the glenoid rim. Instead, the tendon was overlapped and secured only to the capsule [16,17]. Second, to ensure proper tension during subscapularis imbrication, the patient's arm was positioned alongside the body with a 90° elbow flexion and thumb pointing upwards. In this position, the shoulder should achieve a neutral (0°) external rotation [18].

Symeonides identified shortening the stretched subscapularis muscle, creating a double layer of muscle and capsule in front of the joint to form a firm fibrous buttress, and suturing the medial part of the subscapularis over the lateral one to prevent the anterior dislocation of the humeral head during abduction and lateral rotation as three key reasons for the effectiveness, efficacy, and enhanced stability of the modified Putti-Platt procedure [18]. In contrast, the Latarjet procedure utilizes the bony block of the coracoid to extend the glenoid cavity area. During arm abduction and external rotation, the conjoint tendon offers resistance to anterior humeral dislocation. Additionally, the tenodesis effect of the conjoint tendon and the coracoid process resist the deficient anteroinferior aspect of the capsule [19,20].

The study presents some key limitations. This study's non-random sampling, limited age range, single-center design, and quasi-experimental nature limit generalizability and causal inferences. As a first of its kind, these findings require confirmation in larger, well-designed studies.

In summary, while younger patients and males are typically at higher risk of recurrence, this study's results on post-surgery stability, mobility, and function align with existing research. The study also details the Latarjet and modified Putti-Platt procedures, including their goals, potential limitations, and modifications implemented. Interestingly, the study found no significant difference in functional outcomes based on gender and employment status, but social class, education level, and residence did seem to influence outcomes. This suggests that socioeconomic factors may play a role in post-operative recovery and highlights the need for further research on this topic.

## Conclusions

The study found no significant difference in functional outcomes between the Latarjet and modified Putti-Platt procedures for recurrent anterior shoulder instability. The Putti-Platt procedure offers the advantages of shorter operative time and simpler technique, while the Latarjet procedure remains favored by many surgeons. Both procedures can be considered depending on patient characteristics, surgeon preference, and anatomy. Notably, social class, education level, residence, and dislocation type significantly impacted functional outcomes, highlighting the influence of socioeconomic factors. Further research is needed to refine surgical techniques and identify ideal candidates for each procedure.
